# Supplementary material for: Centrosome Amplification Is a Potential Molecular Target in Paediatric Acute Lymphoblastic Leukemia
Source: Cancers (Basel). 2022 Dec 27;15(1):154. doi: 10.3390/cancers15010154 (PMC9818390; doi:10.3390/cancers15010154)
Supplement: Supplementary file 1 [file cancers-15-00154-s001.zip › cancers-2099293-supplementary.pdf]

cancers

MDPI

Article

Centrosome Amplification Is a Potential Molecular Target in Paediatric Acute Lymphoblastic Leukemia

Meiyun Guo[1]†, Jenna Rever[1]†, Phuong N.U. Nguyen[1], Neha M. Akella[1], Gregor S.D. Reid[1,2], and Christopher A. Maxwell[1,2,*].

[1] Department of Pediatrics, University of British Columbia, Vancouver, British Columbia, Canada.
[2] Michael Cuccione Childhood Cancer Research Program, British Columbia Children's Hospital Research, Vancouver, British Columbia, Canada.
* Correspondence: cmaxwell@bcchr.ca
† These authors contributed equally to this work.

Supplementary Tables and Figures

Supplementary Table S1. Sex, subtype, and cytogenetic characterization of stem cells and pediatric B-ALL samples used in the study.

Patient ID	Subtype	Cytogenetics	Gender	Specimen type (%blast)
C03696	Normal	N/A	F	Stem cells (N/A)
C03938	Normal	N/A	M	Stem cells (N/A)
C02571	Normal	N/A	M	Stem cells (N/A)
C01052	Normal	N/A	M	Stem cells (N/A)
C02322	Normal	N/A	M	Stem cells (N/A)
c00652	Re, HyperD	54, XY + chr. X, Y, 9, 14, 21	M	Mononuclear cells (74%)
c00373	Dx, Euploid	N/A	M	N/A (90%)
c00023 r3	Re, Euploid	t(1;5)(q21;q33), t(6;9)(p21;p13), rea(9),t(13 ;17)(q32;q21), t(15 ;16)(q24;q13) t(6;9)	M	Whole bone marrow (90%)
c03632	Dx, HypoD	28, XY	M	Mononuclear cells (93%)
c00023 r2	Re, Euploid	abnormal 2p, 3p, 5q, 6p, 14q, 16q, t(8;9) and t(13;17)	M	Whole bone marrow (96%)
c00115	Re, Euploid	t(16;22), 37% of interphase nuclei have BCR/ABL dual fusion	F	Whole bone marrow (82%)
c00189	Re, HyperD	49-52, XX + chr. X, X, 5, 8, 10, 21, 21 - chr. 20 t(2,8)(p13;p21), del(9)(p21.3), dup (17)(q24.2qter)	F	Whole blood (93%)

c01818	Re, Euploid	del(9)(p21.3)(CDKN2A-, cen9+)	F	Mononuclear cells (98%)
c00269	Re, Euploid	47, XX + chr. 21c	F	Whole bone marrow (99%)
c02100	Re, Euploid	t(9;22)(q34;q11.2)(ABL1+,BCR+,ABL1+)	M	Mononuclear cells (54%)
c00118	Re, Euploid	47, XY + chr. 21c	M	Mononuclear cells (88%)
c00002	Re, Euploid	dic(9;20) del(q9) t(2;5) ins(p5;6) del(q4,q6,q16) inv(3) t(11;15)	M	Whole bone marrow (92%)
C00193	Re, Euploid	dic(9;20) del(CDKN2A,IKFZ1)	M	Mononuclear cells (94%)
C03701	Dx, HyperD	46,X, dup(X)(p21.1p22.3),t(4;9)(q21;q22),dup(6)(q12q14.2)[19]/46,XX[2].ish t(12;21)(p13;q22)(ETV6-,RUNX1+;RUNX1+,ETV6+),del(12)(p13)(ETV6-)	F	Mononuclear cells (94%)
C00189	Re, Euploid	t(2;8)	F	Whole blood (93%)
C00125	Dx, Euploid	del(9p)	F	Whole bone marrow (92%)

Supplementary Table S2. Species, karyotype, origin and cytogenetics of **i**mmortal cell-lines used in this study.

Immortal cell lines	Species	Karyotype	Origin	Cytogenetics
289	Mouse	43 (2N) + chr. 9,12,17	Transgenic Eμ-ret+ mice [25], [26]	RFP/RET fusion gene
RCH-ACV	Human	43 - 50 (2N) + chr. 8	Bone marrow cells taken at relapse from an 8-year-old girl	t(1;19) (q23;p13.3)
380	Human	43 - 47 (2N) - chr. 14	Peripheral blood taken at relapse from a 15-year-old boy	EBNA negative, t(8;14;18) (q24;q32;q21)
RS4;11	Human	46 (2N)	Bone marrow cells taken at relapse from a 32-year-old women	t(4;11) (q21;q23), isochromosome for the long arm of chr.7

Supplementary Table S3. Centrosome clustering inhibitors and chemotherapy drugs used in this study.

Inhibitor Type	Inhibitor	Target	Description
Centrosome Clustering Inhibitor	AZ82 (21898) [20}	KIFC1/HSET	KIFC1 cross-links adjacent microtubules and can focus microtubule minus ends at spindle poles.
	Stattic (S7947) [22]	STAT3	Stattic alters the SH2 domain of STAT3, which acts as transcription factor and also regulates the Stathmin-PLK1 cascade to reduce astral microtubules.

Chemotherapy Drug	Doxorubicin (D1515)	topoisomerase II	Doxorubicin is a widely used anticancer drug, which intercalates in DNA, inhibits topoisomerase II, generates free radicals and induces multipolar spindles.
	Paclitaxel (T7402)	Tubulin	Paclitaxel inhibit microtubules dynamics, arrests cells in mitosis and induces multipolar spindles.

Supplementary Table S4. Genes in TaqMan mouse immune panel.

Gene Type	Gene symbol
**Endogenous control genes**	18S, Gapdh, Hprt1, Gusb
**Immune response associated genes**	Agtr2, Bax, Bcl2, Bcl2l1, C3, Ccl19, Ccl2, Ccl3, Ccl5, Ccr2, Ccr4, Ccr7, Cd19, Cd28, Cd34, Cd38, Cd3e, Cd4, Cd40, Cd40lg, Cd68, Cd80, Cd86, Cd8a, Csf1, Csf2, Csf3, Ctla4, Cxcl10, Cxcl11, Cxcr3, Cyp1a2, Cyp7a1, Edn1, Fas, Fasl, Fn1, Gzmb, H2-Ea, H2-Eb1, Hmox1, Icos, Ifng, Ikbkb, Il10, Il12a, Il12b, Il13, Il15, Il17a, Il18, Il1a, Il1b, Il2, Il2ra, Il3, Il4, Il5, Il6, Il7, Il9, Lrp2, Lta, Nfkb1, Nfkb2, Nos2, Prf1, Ptgs2, Ptprc, Sele, Selp, Ski, Smad3, Smad7, Socs1, Socs2, Stat1, Stat3, Stat4, Stat6, Tbx21, Tgfb1, Tnf, Tnfrsf18, Vcam1, Vegfa, Ace, Icam1, Lif, Ly96, Nfatc3, Nfatc4

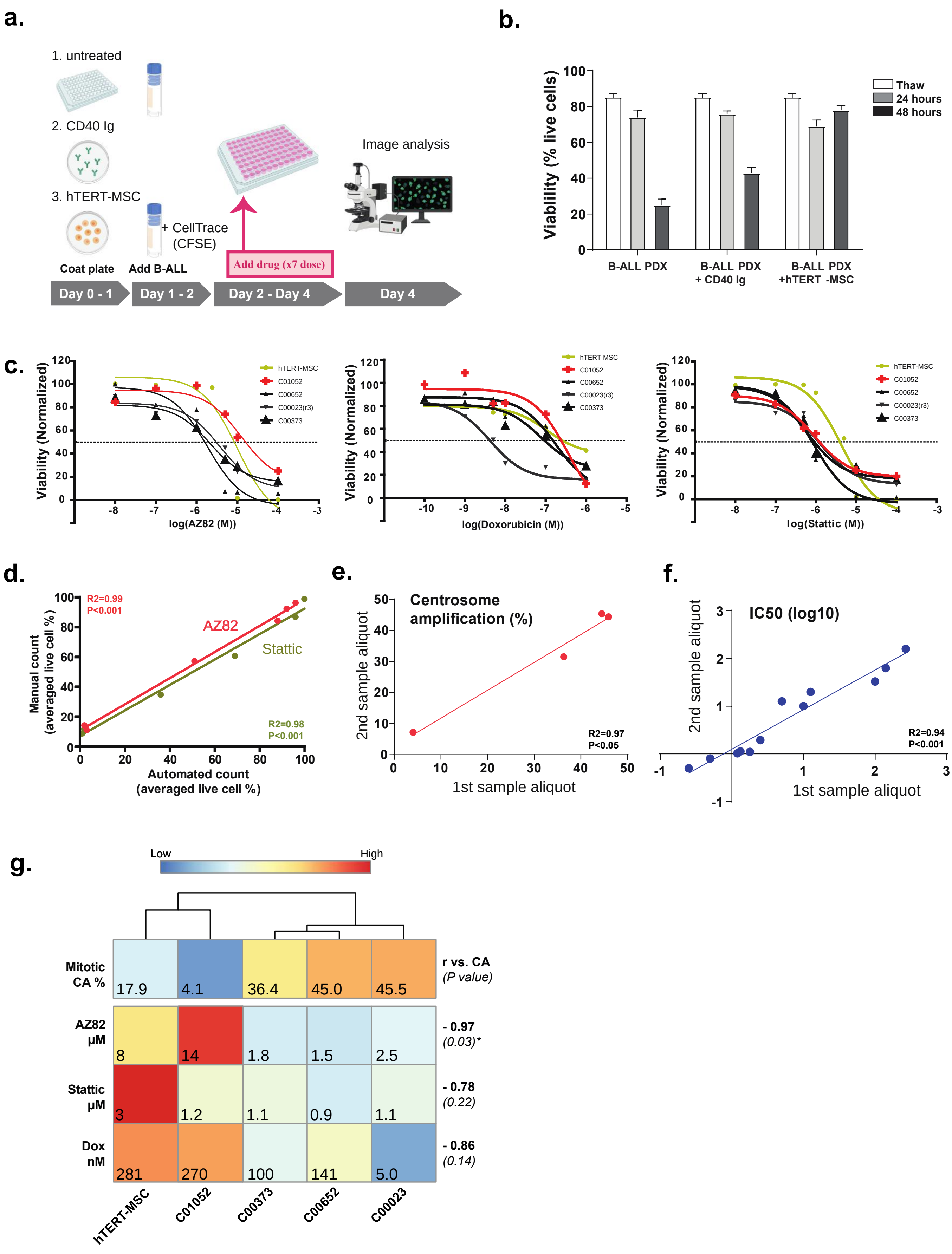

a.
1. untreated
2. CD40 Ig
3. hTERT-MSC
+ CellTrace (CFSE)
Add drug (x7 dose)
Image analysis
Coat plate
Add B-ALL
Day 0 - 1
Day 1 - 2
Day 2 - Day 4
Day 4
b.
Viability (% live cells)
Thaw
24 hours
48 hours
B-ALL PDX
B-ALL PDX + CD40 Ig
B-ALL PDX +hTERT -MSC
c.
Viability (Normalized)
hTERT-MSC
C01052
C00652
C00023(r3)
C00373
log(AZ82 (M))
log(Doxorubicin (M))
log(Stattic (M))
d.
Manual count (averaged live cell %)
Automated count (averaged live cell %)
R2=0.99
P<0.001
AZ82
Stattic
R2=0.98
P<0.001
e.
Centrosome amplification (%)
2nd sample aliquot
1st sample aliquot
R2=0.97
P<0.05
f.
IC50 (log10)
2nd sample aliquot
1st sample aliquot
R2=0.94
P<0.001
g.
Low
High
Mitotic CA %
17.9
4.1
36.4
45.0
45.5
r vs. CA
(P value)
AZ82 μM
8
14
1.8
1.5
2.5
- 0.97
(0.03)*
Stattic μM
3
1.2
1.1
0.9
1.1
- 0.78
(0.22)
Dox nM
281
270
100
141
5.0
- 0.86
(0.14)
hTERT-MSC
C01052
C00373
C00652
C00023

Supplementary Figure S1 Optimization of ex vivo primary cell culture and drug testing **a.** Workflow schematic for *ex vivo* primary cell culture optimization. Conditions of co-culture were: 1. Control untreated. 2. CD40 Ig co-culture. 3. hTERT-MSC co-culture. B-ALL cells were stained with Cell Trace Violet (CFSE) prior to co-culture to ensure distinction from hTERT-MSCs during image analysis. Image created in Biorender.com **b.** Viability of B-ALL patient derived xenograft (PDX) sample cultured in three different conditions (Untreated, CD40 Ig co-culture, hTERT-MSC co-culture). Viability was measured after 0, 24, and 48 hours of culture. **c.** Cell viability normalized to DMSO control in hTERT-MSCs (green), primary bone marrow stem cells control (red), and 3 primary B-ALL samples (black) treated for 48 hours with serial dilutions of centrosome clustering inhibitors (AZ82, Stattic) or a chemotherapy (Doxorubicin). IC50 value is indicated by the dashed line. (mean ± SEM, n=3 experiments). d. Correlation of cell viability measured by high-content image analysis and manual cell counting in AZ82-treated (r2=0.99, p<0.001) and Stattic-treated (R^2=0.98, P<0.001) primary B-ALL cells. **e.** Correlation of biological replicate measurements of centrosome amplification in distinct aliquots from three primary human B-ALL patients and one bone marrow stem cell control (R^2=0.97, p<0.05). **f.** Correlation of biological replicate measurements of IC50 concentrations (log10 μM) for AZ82 and Doxorubicin-treated primary B-ALL cells (R^2=0.94, p <0.001). **g.** Correlation between %CA and IC50 from AZ82, Stattic, or Doxorubicin for 4 primary B-ALL samples and 1 primary bone marrow stem cell sample, and hTERT-MSC cell line. Heatmap columns are ordered by hierarchical clustering based on average linkage, and rows are scaled by Z-score. Pearson r (vs. CA) values (bold) and the p-value (in parentheses) are on the right side (*p<0.05).

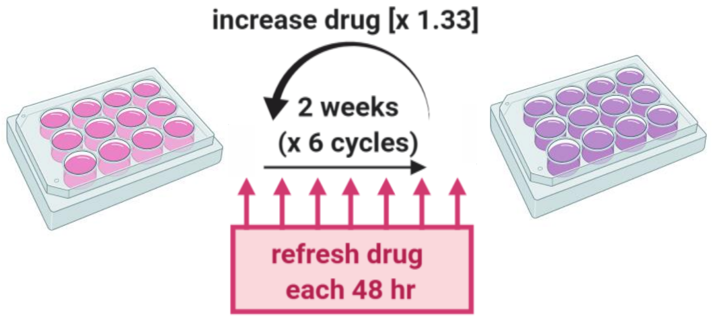

Supplementary Figure S2. Workflow schematic depicting *in vitro* generation of resistant 289 B-ALL cells. 289 cells received increasing concentrations (x1.33 every 2 weeks) of either DMSO or of AZ82 over 4 months. Image created in Biorender.com.
